# Risk Factors for Food Insecurity among Early Childhood Education Providers: Time for a Solution

**DOI:** 10.3390/ijerph21091131

**Published:** 2024-08-27

**Authors:** Dena R. Herman, Skye Shodahl, Holly Wilhalme

**Affiliations:** 1 Department of Family and Consumer Sciences, California State University, 18111 Nordhoff Street, Northridge, CA 91330, USA; 2 Department of Community Health Sciences, UCLA Fielding School of Public Health, 650 Charles E Young Dr. S, Los Angeles, CA 90095, USA; skyesho808@g.ucla.edu; 3 UCLA Division of General Internal Medicine and Health Services Research, 1100 Glendon Ave. Suite 1820, Los Angeles, CA 90095, USA; hwilhalme@mednet.ucla.edu

**Keywords:** early care and education, child care, food insecurity

## Abstract

The COVID-19 pandemic exacerbated challenges in the child care industry, leading to closures and financial strain. Early care and education (ECE) providers faced reduced income, increased debt, and material hardships such as food insecurity. Using survey data collected through the Child Care Resource Center (CCRC), this study examines the association between food insecurity risk, sociodemographic factors, and pandemic-related service changes among ECE providers in California. The results showed that income, race, and increased food costs were significantly associated with a higher risk of food insecurity among ECE providers. Compared to incomes greater than USD 60,000, those earning USD 40,000–USD 49,999 and USD 50,000–USD 59,999 had higher odds of food insecurity (OR: 1.94, 95% CI: 0.683–1.86; OR: 2.12, 95% CI: 0.623–1.81, respectively). Black (OR: 1.89, 95% CI: 1.21–2.94) and multi-racial respondents (OR: 1.71, 95% CI: 1.1–2.65) had higher odds of food insecurity than white respondents. Lastly, respondents experiencing increased food costs had greater odds of food insecurity (OR: 4.52, 95% CI: 2.74–7.45). These findings suggest the need for policies and interventions aimed at increasing food access among vulnerable ECE providers. Such interventions will better protect them from financial shocks and the risk of food insecurity, and will support their crucial role in healthy child growth and development.

## 1. Introduction

The health, growth, and development of young children is a global priority. Recently, the number of children participating in early care and education (ECE) programs has increased globally [1]. The expansion of ECE provisions worldwide is in line with global policy efforts, such as Sustainable Development Goal (SDG) 4, and specifically Target 4.2, which calls for universal access to one year of pre-primary education [2,3,4]. In 2019, nearly 59% of children aged 5 and younger (not enrolled in kindergarten) were in at least one weekly nonparental care arrangement in the U.S. [5]. Within this group, 62% attended center-based care, 38% were in relative care, and 20% received nonrelative care in a private home [5].

Children in the U.S. spent 35.9 h on average per week in nonparental care [6]. Recent data from 196 countries prior to the COVID-19 pandemic showed that ECE enrollment for the population of children between the age of 3 and primary school entry was 54% globally, ranging from 21% in low-income countries to 79% in high-income countries [7]. Considering the number of children attending child care and the significant amount of time children spend in child care, supporting the health and well-being of ECE providers is crucial as their physical and mental health directly impact the quality of care and education they can deliver.

The economic shutdowns and health concerns brought on by the COVID-19 pandemic damaged an already fragile child care industry, putting ECE providers and the children they serve at risk. Between December 2019 and March 2021, approximately 9000 child care centers and 7000 licensed family child care programs closed [8]. A July 2020 survey of ECE providers showed that across settings, child care programs reported incurring additional costs for cleaning supplies (91%), personal protective equipment (76%), staff (72%), and facility changes (27%) [9]. The increased expenses translated to spending, on average, an additional USD 3136 per month for large child care centers, USD 868 per month for small child care centers, and USD 500 per month for family child care homes [9].

The financial strain (i.e., mass closures, decreases in revenue, and increases in operational costs) caused by the pandemic on the child care industry has had severe economic consequences. With near-poverty wages, namely an average rate of USD 13.22 per hour or USD 27,490 per year, ECE providers continue to be underpaid and undervalued [10]. According to a 2021 report, 40% of child care providers had incomes below 185% of the federal poverty guidelines [11]. Other data show that nearly two-thirds (67%) of respondents working in child care centers and three-fourths (76%) of respondents working in family child care homes incurred additional credit card debt for their programs during the pandemic due to limited financial resources [12]. Additionally, one in three ECE providers has experienced at least one material hardship (e.g., food, housing, utilities) during the pandemic, with family/friend/neighbor providers reporting the greatest hardship (43.8%) compared to those in center-based (32.6%) or home-based (32.9%) child care settings [13].

The economic hardships regularly faced by ECE providers were exacerbated by the pandemic and had major consequences on their ability to pay for basic needs including food [14]. Reports of hunger among ECE providers increased during the pandemic from 23% to 29% [15]. Reports of hunger were the highest among child care center teachers (44%) followed by family/friend/neighbor providers (34%), home-based providers (26%), and child care center directors (14%) [15]. During the pandemic, one in three child care providers experienced food insecurity, with approximately 31% reporting running out of money and trying to make their food or food money go further in the last year [11]. Black ECE providers experienced greater rates of high to very high food insecurity (28.5%) compared to white ECE providers (17.8%) [11]. Additionally, higher rates of food insecurity were associated with a lower income and receiving Supplemental Nutrition Assistance Program (SNAP) benefits over the past 12 months [11]. Among ECE providers experiencing high to very high food insecurity, three-fourths were utilizing SNAP benefits [11]. The emotional distress and the economic hardships experienced by ECE providers since the onset of the pandemic underscore the need to understand the risk factors for food insecurity among this vulnerable population. Therefore, the present study examines the relationship between being at risk of food insecurity and the sociodemographic characteristics related to the pandemic among ECE providers. Given the heterogeneity that exists across types of ECE providers, this study also examines differential patterns in risk factors for food insecurity for family ECE providers and license-exempt ECE providers. Identifying and understanding the mechanisms driving food insecurity among ECE providers are necessary for developing effective, sustainable, and relevant solutions to meet the evolving needs of this vulnerable population and the children that they serve.

## 2. Methods

### 2.1. Study Participants

ECE providers were recruited to participate in this study through an invitation distributed by the Child Care Resource Center (CCRC) which serves as a child care resource and referral agency to almost 50,000 children and families in the Antelope Valley, San Fernando Valley, Santa Clarita Valley, and the entirety of San Bernardino County of California. In April 2021, the approximate 2000 ECE providers who comprise this service area were offered participation in a cross-sectional survey to better understand the conditions under which they were working during the COVID-19 pandemic and their access to food resources for themselves and the children they serve. The eligibility criteria included the following: at least 18 years of age, English- or Spanish-speaking, and currently offering child care services. The survey invitation was offered to ECE providers two times during the course of the month at two-week intervals. Participation was voluntary and no incentives were provided. This study was reviewed and approved by the UCLA IRB#21-001394.

### 2.2. Measures

Survey information included age, education, race/ethnicity (white, Black or African American, Asian, Native Hawaiian or Pacific Islander, American Indian or Alaska Native, Hispanic or Latino, and Other), household income, language spoken at home, type of ECE provider (i.e., child care center director, family child care provider, or license-exempt child care provider) [16], number and age of children living at home, and types of meals provided. Definitions for the type of ECE providers as reported in this study are defined by the California (CA) Department of Social Services [17] as follows. A child care center: a licensed child care site that is usually located in a commercial building. Non-medical care and supervision is provided for infant to school-age children in a group setting for periods of less than 24 h. A family child care home: licensed care must be provided in the licensee’s own home. A family child care home reflects a home-like environment where non-medical care and supervision is provided for periods of less than 24 h. License-exempt child care: certain types of providers are exempt from licensure, meaning they can operate legally without a license and are not held to the specific health and safety regulations as those who are licensed. This is the case if they are “subsidized”. The government offers financial assistance, or “subsidies”, to qualified families to help them pay for child care. License-exempt providers who are paid with child care subsidies must register with a particular service and complete a health and safety certification procedure unless they are a close relative. It is important to note that all types of child care listed here may be paid for with subsidies. There are four main groups of child care providers that are license-exempt by the State of CA by obtaining a license. In brief, these are (1) individuals who care for the children of a relative, or who care for the children of one other family in addition to their own children; (2) public as well as private non-profit programs that offer recreational services such as community or recreational centers; (3) businesses that offer limited child care to their clients and customers; and (4) programs that are overseen by state agencies other than Community Care Licensing. For the remainder of this manuscript, child care providers are all those who responded to the study survey questions and identified themselves as being a child care center director or operating a family child care home or license-exempt (subsidized) child care program. We will refer to these types of child care providers as child care center, family child care, and license-exempt child care. The questions to assess service delivery changes related to the pandemic addressed the following: additional resources used to support meal services, increased food costs, and participation in California’s subsidized child care programs. These items were used as predictors for data analyses and were chosen based on previous research [18,19,20].

To identify ECE providers at risk of food insecurity, a validated two-question screening tool based on the U.S. Household Food Security Survey Module called the “Hunger Vital Sign” was used. The question included the addition of the words “and/or my child care” so that family child care and license-exempt child care providers understood that we were referring specifically to the situation as it related to child care and not just their home situation. The two questions asked were as follows: (1) ‘Within the past 12 months I worried whether the food for my household and/or my child care would run out before I got money to buy more’, and (2) ‘Within the past 12 months the food I bought for my household and/or my child care just didn’t last and we didn’t have money to get more’. The responses to these questions included the following: ‘often true’, ‘sometimes true,’ or ‘never true”. In line with established scoring methods, respondents were considered as being at risk of food insecurity if they answered that either or both of the two statements are ‘often true’ or ‘sometimes true’ [21].The Hunger Vital Sign was ideal for this study as it allowed for rapid assessment of food insecurity and was commonly used during the pandemic.

**Figure 1:**
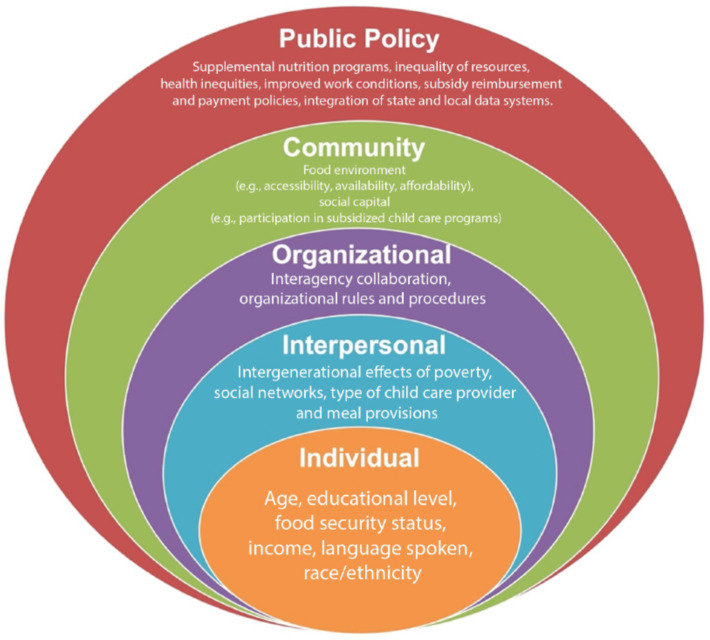
The variables assessed among ECE providers related to the Socioecological Model [22]. Figure adapted from Varela et al. [27].

To guide the statistical analyses, the Social Ecological Model was utilized to assess the strength of the relationships measured across the different system levels that could affect ECE providers’ food security status (see Figure 1 below). Originally developed by Bronfenbrenner [22] and later updated by McLeroy et al. [23], the Social Ecological Model suggests that the health of individuals is affected by the interaction between individuals and the groups they are part of such as their family (e.g., interpersonal), the community, and their physical, social (e.g., organizational), and political (e.g., policy) environments [21,24,25]. This framework was also included in the 1947 World Health Organization’s Constitution to encompass an expanded vision of health that includes physical, mental, and social well-being [26]. Using this framework, the variables indicated above are included in the levels of this model below with an asterisk. Variables identified in the literature as relevant are also mentioned and discussed further in the Discussion Section. The levels are as follows: (1) Individual: age*, educational level*, food security status*, income*, language spoken*, race/ethnicity*; (2) Interpersonal: intergenerational effects of poverty, social networks, type of child care provider*, and meal provisions*; (3) Organizational: interagency collaboration and organizational rules and procedures; (4) Community: food environment (e.g., accessibility, availability*, affordability*) and social capital (e.g., participation in subsidized child care programs*); (5) Public Policy: supplemental nutrition programs*, inequality of resources, health inequities, improved work conditions, subsidy reimbursement and payment policies, and the integration of state and local data systems.

### 2.3. Statistical Analysis

Descriptive statistics were calculated to explore the distribution of the data among participants. The Chi-square test was conducted to compare participant sociodemographic characteristics and changes in service delivery related to the pandemic between ECE provider types. To determine variables that were associated with being at risk of food insecurity, a multivariate logistic regression model predicting the Hunger Vital Sign outcome was constructed. First, a bivariate analysis was conducted using the Chi-square test to determine individual variables that were associated with the outcome from sociodemographic characteristics (i.e., age, education, race/ethnicity, household income, language spoken at home, and type of ECE provider) and changes in service delivery related to the pandemic (i.e., meal-type provision at child care program, additional resources used to support meal services, increased food costs, and participation in California’s subsidized child care programs which provide subsidies, or financial assistance, to help families afford child care). Variables that were significant at *p* < 0.05 were included in the model. Additionally, two variables “experienced increase food costs…” and “experienced food shortage” were entered into the model based on theoretical assumptions. To identify differential patterns in risk factors for food insecurity across ECE provider type, stratified logistic regression analyses were conducted separately for family child care providers and license-exempt child care providers. Child care center directors were dropped from the analysis at the stage of bivariate analyses due to the lack of significance at *p* < 0.05. To increase power and protect participant anonymity, the levels for several of the predictor variables were collapsed. We aggregated categories for the following predictor variables: age, race, income, and language spoken at home. Age categories were consolidated by combining those under 18 with the 18–29 group. In the case of race, we merged Asian, Native Hawaiian or Pacific Islander (AAHNPI), American Indian or Alaska Native (AI/AN), and other categories into a single category. Additionally, bi-racial and multi-racial designations were combined into a single category. For income, the categories of USD 60,000–USD 69,999, USD 70,000–USD 79,999, and >USD 79,999 were combined into a single category. Finally, language spoken at home was simplified by combining the “other” and “bi-lingual” categories.

We constructed the following empirical model to evaluate the relationship between food insecurity and the following independent variables:

Logit (P (Y = 1)) = α + β_1_x_1_ + β_2_x_2_ + …. + β_n_x_n_,

where:

Y = food insecurity;
α is the intercept;
x1 = income [coded in the following categories: <USD 20,000 (reference category); USD 20,000–USD 29,999; USD 30,000–USD 39,999; USD 40,000–USD 49,999; USD 50,000–USD 59,999; and ≥USD 60,000];
x2 = race [coded in the following categories: white (reference category); Black, Hispanic, AAHNPI/AIAN, and other; 2 or more races];
x3 = experiencing increases in food costs (coded in the following categories: yes or no);
x4 = types of child care provider [coded in the following categories: license-exempt child care (reference category); family child care];
x5 = educational attainment [coded in the following categories: 4-year college degree (reference category); less than a high school degree; high school degree or graduate equivalency degree (GED); attended college, but have not graduated; two-year college degree];
x6 = age [coded in the following categories: 60 and over (reference category); <18–29 y; 30–39 y; 40–49 y; 50–59 y];
x7 = language spoken at home [coded in the following categories: English (reference category); Spanish; other; and bi-lingual];
x8 = type of meal provision at child care program [coded in the following categories: Other (reference category); breakfast, lunch, dinner, supper, snack(s); breakfast, lunch, snack(s)];
x9 = utilization of additional resources to support meal services (coded in the following categories: yes, no);
x10 = participation in California’s child care programs (coded in the following categories: yes, no).

To assess model fit, the Hosmer–Lemeshow goodness-of-fit test was used. Pseudo R-squared was calculated to evaluate the proportion of variance in the outcome variable explained by the model. All analyses were conducted using IBM SPSS Statistics software [28]. *p*-values < 0.05 were considered statistically significant

## 3. Results

### 3.1. Univariate Analyses

Table 1 describes the sociodemographic and lifestyle characteristics of the study sample by ECE provider type. Of the 1423 survey respondents, approximately 76% (*n* = 1059) reported being at risk of food insecurity. Among the 1123 family ECE providers, 75% were reported to be at risk of food insecurity. The majority were aged 50-59 years (33.6%), attended college but did not graduate (31.4%), identified as either Hispanic (25.8%) or Black (25%), had a household income of less than USD 20,000 in the year 2020 (26%), spoke English at home (66.2%), and had children living at home, the majority of whom were 6-18 years of age (40.1%). Nearly half of family child care providers participated in California’s subsidized child care programs (49.7%). Within their household or child care program, the majority of family child care providers experienced an increase in food costs since the start of the pandemic (94.2%), sometimes worried whether food for their household and/or their child care would run out before they earned money to buy more (51%) and sometimes (44.3%) worried whether the food they bought for their household and/or their child care just would not last and they would not have money to buy more. Within their child care program, the majority of family child care providers provided breakfast (91.6%), lunch (95.9%), and snacks (92.3%). To help with the costs of meal provisions, approximately 26% sought support from the Child and Adult Care Food Program, 29% received free meals from schools, child care centers, or other places, and 15% received free groceries from churches, food banks, or other places.

Among the 300 license-exempt child care providers who responded, approximately 78% reported being at risk of food insecurity. The majority were aged 50–59 years old (28.4%), had at least a high school degree or GED (40.2%), identified as either Hispanic (29.3%) or Black (30.3%), had a household income of less than USD 20,000 in the year 2020 (41.9%), spoke English at home (77.2%), and had no children living at home (43.3%). Nearly 41% of license-exempt child care providers participated in California’s subsidized child care programs. Within their household or child care program, the majority of license-exempt child care providers experienced an increase in food costs since the start of the pandemic (92.7%), sometimes worried whether food for their household and/or their child care would run out before they earned money to buy more (54.8%) and sometimes worried whether the food they bought for their household and/or their child care just would not last and they would not have money to buy more (43.6%). Within their child care program, the majority of license-exempt child care providers provided breakfast (83.3%), lunch (91%), and snacks (93.7%). To help with the costs of meal provisions, approximately 4% sought support from the Child and Adult Care Food Program, 35% received free meals from schools, child care centers, or other places, and 23% received free groceries from churches, food banks, or other places.

The results of the chi-square analyses (Table 1) identified differences in sociodemographic and lifestyle characteristics between ECE provider types. Family child care providers were more likely to be 50–59 y of age compared to license-exempt child care providers (33.6% vs. 28.4%), more likely to attend college (31.4% vs. 29.1%), more likely to report being white (18.3% vs. 11.1%), less likely to have incomes <USD 20,000 (26% vs. 41.9%), more likely to be Spanish speakers (21% vs. 18.5%), more likely to have children older than 9 years of age at home (14.9% vs. 10.3%), more likely to serve breakfast, lunch, and dinner in their child care programs (breakfast: 91.6% vs. 83.3%; lunch: 95.9% vs. 91%; dinner: 73.7% vs. 64%), more likely to utilize the CACFP program to support meal provisions in their child care programs (25.7% vs. 3.7%), and more likely to participate in subsidized child care programs (49.7% vs. 41.2%).

### 3.2. Multivariate Analyses

Table 2 shows the results from the logistic regression analyses estimating the association between respondents’ report of being at risk of food insecurity and their sociodemographic characteristics. The model also included variables assessing changes in service delivery related to the pandemic across ECE provider type which were entered based on theoretical assumptions.

The results indicate that income (*p* < 0.01), race (*p* < 0.05), and increases in food costs for their household and/or child care program (*p* < 0.01) remained significant factors associated with an increased risk of being food-insecure in the multivariate model. Compared to respondents with an income greater than USD 60,000, the odds of being at risk of food insecurity were higher for those with an income between USD 40,000 and 49,999 (OR: 1.94, 95% CI: 0.683–1.86) and between USD 50,000 and 59,999 (OR: 2.12, 95% CI: 0.623–1.81). There were no significant differences in the risk of food insecurity between respondents with an income greater than USD 60,0000 and respondents with an income less than USD 20,000 and equal to USD 20,000–USD 29,999 (OR: 1.13, 95% CI: 0.683–1.86) or USD 30,000–USD 39,999 (OR: 1.29, CI: 0.824–2.03). Compared to white respondents, the odds of being at risk of food insecurity were higher for Black (OR: 1.89, 95% CI: 1.21–2.94) and multi-racial respondents (OR: 1.71, 95% CI: 1.1–2.65). There were no significant differences in the risk of food insecurity between white respondents and respondents who identified as Hispanic (OR: 1.18, CI:.755–1.85), Asian American, Native Hawaiian/Pacific Islander or American Indian/Alaska Native, or another race (OR: 1.78, CI: 0.848–3.74). Respondents who reported increases in food costs for their household and/or child care program since the pandemic had an increased odds of being at risk of food insecurity compared to respondents who did not experience increases in food costs (OR: 4.52 95% CI: 2.74–7.45). The association between food insecurity and all other analytic variables selected for inclusion in the model did not show a statistically significant relationship (see Table 2).

Based on the Hosmer–Lemeshow goodness-of-fit test, the model fit the data well (χ^2^ = 8.70, *p* > 0.05). The model exhibited limited explanatory capacity with Cox and Snell and Nagelkerke pseudo R-squared values of 0.062 and 0.093, respectively.

To identify differential patterns in risk factors for food insecurity across caregiver type, stratified logistic regression analyses were conducted separately for family child care providers and license-exempt child care providers. Due to the low sample size within the stratum for license-exempt child care providers, little to no differences in risk factors for food insecurity were identified between the two ECE provider types. For this reason, the results of the stratified analyses were not reported.

## 4. Discussion

ECE providers, of whom the majority are women and racial/ethnic minorities, are at an increased risk of food insecurity due to their low wages. This situation becomes more dire during times of increased economic strain such as during the COVID-19 pandemic. In this study of 1423 ECE providers (1123 family child care providers and 300 license-exempt child care providers), at least three-quarters experienced an increased risk of food insecurity during this period. Moreover, these ECE providers experienced an increase in food costs, which led to 4.5 times higher odds for the risk of being food-insecure.

Compared with ECE providers with incomes of USD 60,000 or higher, the risk of food insecurity was approximately two times as high for ECE providers with incomes between USD 40,000 and 49,999 and those with incomes between USD 50,000 and 59,999. However, the risk of food insecurity was not significantly higher for those with incomes less than USD 40,000. One explanation for this finding is that individuals with higher income brackets may not qualify for public assistance programs such as SNAP, while those with lower income brackets can. This may create a gap where those with higher incomes struggle without the support that those who have fewer resources receive. For those ECE providers who identified as Black or multi-racial, the odds for the risk of being food-insecure were almost twice as high compared to ECE providers from other ethnic minorities.

Other studies have confirmed the economically precarious nature of child care providers, which typically constitutes poverty-level wages, a lack of resources, and inadequate public funding [29,30]. This is also true of ECE providers nationally whose annual pay remains almost in the bottom percentile compared to all other occupations according to the Early Childhood Workforce Index [31]. In a study examining the relationship between wage-setting policies and food insecurity, the authors demonstrated that full-time, low-wage workers from 139 countries experienced lower rates of food insecurity when supported by more generous wage-setting policies [32]. As with our study, the results from Linnan et al. [33] demonstrate that 42% of those who worked in child care centers in North Carolina reported living below the federal poverty limit and 66% had an Associate’s degree or less. As explained in a recent report, early childhood educators are poorer and “less organized”—meaning that they are less likely to work in formalized work unions—than other workforce groups and more likely to be women of color [30]. These conditions have been shown to undermine their well-being and create detrimental financial conditions that last well into retirement. Together, these conditions may jeopardize their ability to effectively care for the children for whom they are responsible [34,35].

In this study, more than three-quarters of ECE providers reported being at risk of FI, which is almost double that of other studies reporting rates of FI at about 41% [36]. Swindle et al. [37] found that about 28% of ECE providers experience FI as children and that this influences how they respond to perceptions of FI among the children they care for, especially in the absence of specific guidance or dedicated training. Many of these problems were worsened by the COVID-19 pandemic and included the fact that many ECE programs were forced to shut down, reduce their hours, and/or reduce their compensation [38,39]. Furthermore, as in our study, ECE providers nationally have reported that they lacked resources to purchase more food and were forced to rely on federal nutrition assistance programs not only to serve the children in their programs but also for themselves [40]. These types of financial instability issues may have health repercussions for the ECE providers themselves and result in reductions in the number of meals served, further destabilizing child care businesses and their workers [41].

Financial strains were felt the deepest by women during the COVID-19 pandemic, and women comprise the overwhelming majority of the child care workforce. In this study, these strains were experienced at higher rates by child care providers who were Black and multi-racial. One explanation for the poor compensation and lack of benefits for ECE providers and workers today—especially those of color—is that longstanding legislation intentionally excluded domestic workers and, with that, a number of Black women and other women of color from key labor protections [42]. Nationally, research shows that ECE workers who are Black earn lower wages than their peers even after controlling for educational attainment [43]. ECE workers of color are also more likely to hold lower wage positions such as assistant teachers or aide positions, which further exacerbates these injustices [43]. Black women face increased social inequities and psychosocial stress in general, and during the COVID-19 pandemic, these were heightened. Black women are also more likely than their white counterparts to hold roles as “essential workers,” which increased their risk for COVID-19 [44,45]. Together, financial insecurity and psychosocial stress can hinder this population of child care workers’ ability to mitigate and manage these stressors, which can lead to adverse health events including increased rates of anxiety and depression.

While this study did not measure these health outcomes, there is a body of research connecting poor mental health and food insecurity among female populations with low incomes in smaller and national studies [46,47,48,49]. Loh et al. [36] showed that among ECE workers in Washington and Texas, there was a 4.95 times higher risk of depression among those reporting very low and low food security compared to those who were food-secure. Very low food security indicates that households may be skipping meals or reducing their intake because they could not afford to eat enough food, while low food security indicates that household may be reducing the variety and quality of the foods that they eat [50]. Previous studies have also shown this relationship among child care workers [14,33]. Three-quarters of both family child care providers and license-exempt child care providers in this study experienced an increased risk of food insecurity, also putting them at a higher risk of emotional distress, potentially resulting in outcomes such as anxiety or depression.

### 4.1. Limitations

This study had several limitations. Because the original intent of data collection was to support California legislation for increased SNAP provisions for ECE providers, respondent burden was considered and only a limited number of variables were assessed to not cause further undue stress on the population of interest. As such, information that might have also assessed mental health outcomes was not collected. In addition, while the survey was sent on two separate occasions to all 2000 ECE providers that comprise the CCRC child care resource and referral agency, no incentives were provided. As such, the resulting sample is a convenience sample and may represent those ECE providers who were most interested in sharing their situation. Moreover, if more providers could have been included in this study, this would have increased the generalizability of our findings. However, it must be noted that while the study population is racially and ethnically diverse, the sampling strategy does not allow us to make generalizations to how these results may affect other similar populations. To gain a more comprehensive understanding of the complex factors contributing to food insecurity among ECE providers, future studies should employ mixed-method approaches that integrate quantitative surveys with qualitative interviews or focus groups. Finally, to increase statistical power, we chose to aggregate categories for age, race, income, and language spoken at home. This may have decreased our ability to identify important information with respect to subgroups that deserve additional attention for the hardships they are encountering. However, in working with the CCRC, we also ensured that we would protect participant privacy and anonymity and therefore decided on this statistical approach in the interest of our study sample. Nevertheless, the resulting sample did provide a relatively large sample of very hard-to-reach license-exempt child care providers for whom very little data exist and a sizable number of family child care providers. Although the data set is limited in the number of variables included, the data provide up-to-date information on a critical segment of the workforce about whom little is known, especially with respect to food insecurity.

### 4.2. Public Health Implications

Because ECE providers’ economic situations are precarious even under usual conditions, sources of more consistent support should be sought to help stabilize the availability of resources, particularly for food. In the global setting, the call to action has focused on taking a person-centered approach. This initiative has focused on meeting the needs of individuals to facilitate integration across services and sectors for coordinated service delivery to improve outcomes for children and families [51]. In the U.S., better access and utilization by ECE providers of government nutrition assistance programs may be a first step on the path to more consistent food access. The Child and Adult Care Food Program (CACFP) provides reimbursement for nutritious meals and snacks to income-eligible children at participating child care sites and also provides free nutrition training and access to commodity foods [52]. All of these services could work to support ECE providers and the children they serve to lower the identified risks of becoming food-insecure [53]. Lee et al. [54] found that among 16 CACFP independent operating centers, the benefits of CACFP participation included reimbursement for food, supporting communities with low incomes, and the provision of healthy food guidelines. While some barriers were also mentioned such as paperwork, administrative reviews, communication, inadequate reimbursement, staffing, nutrition standards, training needs, eligibility determination, and technological challenges, sponsored centers were able to resolve a number of these issues with the technical assistance they received.

Another means for increasing access to food resources is to increase the benefits available to ECE providers and workers through the Supplemental Nutrition Assistance Program (SNAP). SNAP is the largest food safety net in the United States and in 2022 served almost 42 million people and 12% of the population nationally. In California, SNAP is known as CalFresh and participation rates mirrored those nationally, with 12% of those eligible participating [55]. Additional SNAP benefits were made available during the COVID-19 pandemic, but emergency allotments have now ended.

With respect to the Social Ecological Model, we have demonstrated that relationships exist across the different levels of systems that ECE providers access and their reports of food insecurity [22,23]. These include the relationship between food insecurity and individual-level factors (i.e., race/ethnicity and income) and community-level factors (e.g., food environment including food costs and food resources). Community interventions that may improve or stabilize food insecurity for ECE providers beyond higher participation rates in governmental nutrition programs such as CACFP and SNAP include greater access to local food sources. These may include food pantries, community meal distribution sites, and community gardens and fridges. Transportation to these sites may be another social determinant that needs addressing to improve food security for ECE providers and workers [56]. While this study did not measure factors on the interpersonal or public policy levels, research shows that intergenerational poverty has had a long-lasting effect on ECE providers’ ability to earn a living wage, keeping them and their families at near-poverty levels [30]. Public policy efforts may be the best route to breaking this chain of effects [42]. Policy efforts that have been proven to be effective with other populations include higher participation rates in SNAP and other nutrition assistance programs and improving equality in financial and other health resources [40].

These more targeted approaches to mitigate food insecurity among ECE providers should be used in conjunction with strategies proposed by the National Academies of Sciences, Engineering, and Medicine to address the impact of COVID-19 on the ECE sector [57]. These strategies include increasing compensation, improving overall work conditions, changing subsidy reimbursement and payment policies (e.g., basing subsidy rates on enrollment rather than attendance), reducing the rates of closures by directing recovery funds to the ECE sector, and supporting the integration of state and local data systems to increase access to real-time information on ECE providers and assist with rapid response planning [57].

## 5. Conclusions

This study found that during the COVID-19 pandemic, ECE providers who were racial and ethnic minorities had a higher risk of food insecurity, regardless of whether they were family child care providers or license-exempt child care providers. Higher food costs were also associated with a higher risk of food insecurity for these populations. Ongoing local, national, and international policies along with ECE site-specific interventions to increase access to additional food resources may help protect this critical yet vulnerable population from episodic financial shocks. Multilevel policies and programs may also prove critical to protecting ECE providers from related risks of food insecurity and may support their crucial role in healthy child growth and development.

## Figures and Tables

**Table 1:** Sociodemographic and lifestyle characteristics of study sample by early care and education (ECE) provider type ^1^ (*n* = 1423).

	Family Child Care Provider (*n* = 1123)	License-Exempt Child Care Provider (*n* = 300)	*p*-Value
	*n* (Row%)	*n* (Row%)	
Age (years)			0.001 **
Under 18	14 (1.3)	5 (1.7)	
18–29	65 (5.8)	39 (13)	
30–39	178 (15.9)	50 (16.7)	
40–49	266 (23.8)	67 (22.4)	
50–59	375 (33.6)	85 (28.4)	
60 and over	218 (19.5)	53 (17.7)	
Education			<0.001 **
Less than a high school degree	148 (13.4)	36 (12.2)	
High school degree or graduate equivalency degree	289 (26.1)	119 (40.2)	
Attended college, but have not graduated	348 (31.4)	86 (29.1)	
Two-year college degree	161 (14.5)	30 (10.1)	
Four-year college degree	162 (14.6)	25 (8.4)	
Race			0.004 **
White	204 (18.3)	33 (11.1)	
Black	278 (25)	90 (30.3)	
Asian	34 (3.1)	2 (0.7)	
Native Hawaiian or Pacific Islander (NW/PI)	2 (0.2)	1 (0.3)	
American Indian or Alaska Native (AI/AN)	2 (0.2)	1 (0.3)	
Hispanic	287 (25.8)	87 (29.3)	
Other	18 (1.6)	0 (0)	
Bi-racial	278 (25)	79 (26.6)	
Multi-racial	9 (0.8)	4 (1.3)	
Household income			<0.001 **
<USD 20,000	272 (26)	122 (41.9)	
USD 20,000–USD 29,999	190 (18.1)	64 (22)	
USD 30,000–USD 39,999	152 (14.5)	39 (13.4)	
USD 40,000–USD 49,999	111 (10.6)	21 (7.2)	
USD 50,000–USD 59,999	86 (8.2)	18 (6.2)	
USD 60,000–USD 69,999	53 (5.1)	9 (3.1)	
USD 70,000–USD 79,999	68 (6.5)	12 (4.1)	
>USD 79,999	115 (11)	6 (2.1)	
Language spoken at home			<0.001 **
English	735 (66.2)	230 (77.2)	
Spanish	233 (21)	55 (18.5)	
Bi-lingual	103 (9.3)	11 (3.7)	
Other ^5^	40 (3.6)	2 (0.7)	
Age of children living at home ^1^			
No children	439 (39.1)	130 (43.3)	0.183
Under 6 years old	219 (19.5)	68 (22.7)	0.225
6–18 years old	450 (40.1)	112 (37.3)	0.389
9 years or older	167 (14.9)	31 (10.3)	0.044 *
Type of meal provision at child care ^2^			
Breakfast	1029 (91.6)	250 (83.3)	<0.001 **
Lunch	1077 (95.9)	273 (91)	<0.001 **
Dinner	828 (73.7)	192 (64)	<0.001 **
Snacks	1037 (92.3)	281 (93.7)	0.436
Utilization of additional resources to support meal provision			
Child and Adult Care Food Program	289 (25.7)	11 (3.7)	<0.001 **
Free groceries (from churches, food banks, etc.)	169 (15)	68 (22.7)	0.002 **
Free meals (from schools, child care centers, or other locations)	323 (28.8)	106 (35.3)	0.028 *
Experienced increased costs of food for household and/or child care			
Yes	1047 (94.2)	278 (92.7)	0.342
Participation in subsidized child care programs ^6^			
Yes	543 (49.7)	121 (41.2)	0.009 *
Worried about food shortage in household and/or child care ^3^			0.319
Often True	240 (21.8)	66 (22.1)	
Sometimes True	562 (51)	164 (54.8)	
Never True	301 (27.3)	69 (23.1)	
Experienced Food Shortage And Inability To Buy More For Household And/Or Child Care ^4^			0.542
Often True	134 (12.2)	43 (14.4)	
Sometimes True	478 (43.5)	130 (43.6)	
Never True	487 (44.3)	125 (41.9)	

^1^ The definitions for early care and education (ECE) providers are as follows. A family child care home: licensed care must be provided in the licensee’s own home. A family child care home reflects a home-like environment where non-medical care and supervision is provided for periods of less than 24 h. License-exempt child care: certain types of providers are exempt from licensure, meaning they can operate legally without a license. License-exempt providers who are paid with child care subsidies must register with a specific governmental service and complete a health and safety certification procedure unless they are a close relative. ^2^ The respondent was asked to select more than one response. ^3^ Full statement provided in the survey: ‘Within the past 12 months I worried whether the food for my household and/or my child care would run out before I got money to buy more’. ^4^ Full statement provided in survey: ‘Within the past 12 months the food I bought for my household and/or my child care just didn’t last and we didn’t have money to get more’. ^5^ Other includes Armenian, Dari, Farsi, Ibo, Korean, Russian, and Sinhalese. ^6^ The survey was sent to the following types of child care providers: child care center directors, family day care homes and license-exempt child care programs. For this question, some of the programs that had originally indicated that they were “subsidized” license-exempt child care programs reported in this question that they did not participate in that program. * Significant at <0.05. ** Significant at <0.01.

**Table 2:** Results of logistic regression predicting being at risk of food insecurity among early care and education (ECE) providers (*n* = 1246) ^1^.

Predictor	Coefficient	OR (S.E.)	95% CI	*p*
Income (>USD 60,000 = ref category)	-	-	-	0.005 *
<USD 20,000	0.059	1.06 (0.272)	1.38–3.26	0.829
USD 20,000–29,999	0.120	1.13 (0.256)	1.23–3.03	0.639
USD 30,000–39,999	0.257	1.29 (0.230)	0.824–2.03	0.264
USD 40,000–49,999	0.660	1.94 (0.229)	0.683–1.86	0.004 *
USD 50,000–59,999	0.751	2.12 (0.220)	0.623–1.81	<0.001 *
Race (white = ref category)	-	-	-	0.017 **
Black	0.634	1.89 (0.226)	1.21–2.94	0.005 *
Hispanic	0.167	1.18 (0.228)	0.755–1.85	0.466
AANHPI, AI/AN, Other ^2^	0.577	1.78 (0.379)	0.848–3.74	0.128
2 or more races	0.534	1.71 (0.225)	1.1–2.65	0.018 **
Experiencing increases in food costs	1.51	4.52 (0.255)	2.74–7.45	<0.001 *
Type of child care provider (license-exempt) = ref category) ^3^	−0.035	0.966 (0.180)	0.679–1.37	0.845
Educational attainment (four-year college degree = ref category)	-	-	-	0.57
Less than a high school degree	−0.454	0.635 (0.305)	0.349–1.16	0.137
High school degree or GED ^4^	−0.245	0.783 (0.248)	0.481–1.27	0.323
Attended college, but have not graduated	−0.283	0.754 (0.236)	0.475–1.2	0.231
Two-year college degree	−0.375	0.688 (0.267)	0.407–1.16	0.16
Age (60 and over = ref category)	-	-	-	0.751
<18–29	0.986	0.986 (0.301)	0.547–1.78	0.963
30–39	1.14	1.14 (0.241)	0.71–1.83	0.587
40–49	0.898	0.898 (0.210)	0.594–1.36	0.609
50–59	1.13	1.13 (0.200)	0.761–1.67	0.549
Language spoken at home (English = ref category)	-	-	-	0.155
Spanish	0.696	0.696 (0.205)	0.466–1.04	0.077
Other and bi-lingual	1.10	1.1 (0.242)	0.683–1.76	0.699
Type of meal provision at child care program (Other = ref category)	-	-	-	0.372
Breakfast, lunch, dinner/supper, snack(s)	0.830	0.830 (0.214)	0.546–1.26	0.384
Breakfast, lunch, snack(s)	0.716	0.716 (0.239)	0.449–1.14	0.163
Utilization of additional resources to support meal service ^5^	0.861	0.861 (0.144)	0.648–1.14	0.298
Participation in California’s subsidized child care programs	−0.150	0.808 (0.150)	0.602–1.09	0.156

^1^ The number of respondents in the analytic sample who were categorized as at-risk of food insecurity, based on the validated two-question Hunger Vital Sign, was 948 (76.1%). ^2^ AAHNPI = Asian, Native Hawaiian or Pacific Islander; AI/AN = American Indian or Alaska Native. ^3^ The comparison category to license-exempt child care providers (reference category) is family child care providers. ^4^ GED = graduate equivalency degree. ^5^ Respondents identified as using at least one of the following resources to support meal services in their child care program: (1) Child and Adult Care Food Programs, (2) free groceries (from churches, food banks, etc.), or (3) free meals (from schools, child care centers, or other locations). * Significant at *p* < 0.05. ** Significant at *p* < 0.01.

## Data Availability

The data presented in this study are available upon request from the corresponding author due to privacy, legal and ethical concerns.

## References

[1] UNESCO Institute for Statistics (2019). How to Produce and Use the Global and Thematic Education Indicators.

[2] (2017). Revised List of Global Sustainable Development Goal Indicators.

[3] (2015). Transforming Our World: The 2030 Agenda for Sustainable Development.

[4] Von Suchodoletz A., Lee D.S., Henry J., Tamang S., Premachandra B., Yoshikawa H. (2023). Early Childhood Education and Care Quality and Associations with Child Outcomes: A Meta-Analysis. PLoS ONE.

[5] U.S. Department of Education, National Center for Education Statistics Early Childhood Program Participation: 2019 (NCES 2020-075REV), Table 1. https://nces.ed.gov/fastfacts/display.asp?id=4.

[6] U.S. Department of Education, National Center for Education Statistics Early Childhood Program Participation Survey of the National Household Education Surveys Program (ECPP-NHES:2016). https://nces.ed.gov/programs/digest/d19/tables/dt19_202.30.asp.

[7] McCoy D.C., Cuartas J., Behrman J., Cappa C., Heymann J., López Bóo F., Lu C., Raikes A., Richter L., Stein A. (2021). Global Estimates of the Implications of COVID-19-related Preprimary School Closures for Children’s Instructional Access, Development, Learning, and Economic Wellbeing. Child Dev..

[8] Child Care Aware (2022). Demanding Change: Repairing Our Child Care System.

[9] National Association for the Education of Young Children (2021). Holding On Until Help Comes A Survey Reveals Child Care’s Fight to Survive.

[10] U.S. Bureau of Labor Statistics Occupational Outlook Handbook: Childcare Workers. https://www.bls.gov/ooh/personal-care-and-service/childcare-workers.htm.

[11] Dynia J.M., Koury A.J., Bates R.A., McGinnis C.P. (2021). Food Insecurity in a Nationally Representative Sample of Child Care Workers [White Paper].

[12] National Association for the Education of Young Children (2021). Progress and Peril: Child Care at a Crossroads.

[13] (2021). Center for Translational Neuroscience. Who Is Providing for Child Care Providers?.

[14] Otten J.J., Bradford V.A., Stover B., Hill H.D., Osborne C., Getts K., Seixas N. (2019). The Culture Of Health in Early Care and Education: Workers’ Wages, Health, and Job Characteristics. Health Aff..

[15] Center for Translational Neuroscience (2021). Who Is Providing for Child Care Providers? Part 2.

[16] California Department of Social Services Types of Child Care in California. https://www.cdss.ca.gov/inforesources/child-care-licensing/resources-for-parents.

[17] Child Care Law Center (2022). Know the Law About License Exempt Care in California.

[18] Gould E. (2015). Child Care Workers Aren’t Paid Enough To Make Ends Meet.

[19] Lessard L.M., Wilkins K., Rose-Malm J., Mazzocchi M.C. (2020). The Health Status of the Early Care and Education Workforce in the USA: A Scoping Review of the Evidence and Current Practice. Public Health Rev..

[20] Scott K., Looby A.A., Hipp J.S., Frost N. (2017). Applying an Equity Lens to the Child Care Setting. J. Law Med. Ethics.

[21] Hager E.R., Quigg A.M., Black M.M., Coleman S.M., Heeren T., Rose-Jacobs R., Cook J.T., De Cuba S.A.E., Casey P.H., Chilton M. (2010). Development and Validity of a 2-Item Screen to Identify Families at Risk for Food Insecurity. Pediatrics.

[22] Bronfenbrenner U. (1977). Toward an Experimental Ecology of Human Development. Am. Psychol..

[23] McLeroy K.R., Bibeau D., Steckler A., Glanz K. (1988). An Ecological Perspective on Health Promotion Programs. Health Educ. Q..

[24] Sallis J.F., Owen N. (2015). Health Behavior: Theory, Research, and Practice. Ecological Models of Health Behavior.

[25] Wallerstein N., Duran B. (2003). The Conceptual, Historical and Practical Roots of Community Based Participatory Research and Related Participatory Traditions. Community Based Participatory Research for Health. From Process to Outcomes.

[26] Sharp W.R. (1947). The New World Health Organization. Am. J. Int. Law.

[27] Varela E.G., McVay M.A., Shelnutt K.P., Mobley A.R. (2023). The Determinants of Food Insecurity among Hispanic/Latinx Households with Young Children: A Narrative Review. Adv. Nutr..

[28] SPSS (2007). Statistical Package for the Social Sciences.

[29] Gould E., Blaire H. (2020). Who’s Paying Now? The Explicit and Implicit Costs of the Current Early Care and Education System.

[30] McLean C., Austin L.J.E., Whitebook M., Olso K.L. (2020). Early Childhood Workforce Index 2020.

[31] U.S. Bureau of Labor Statistics Occupational Employment and Wage Statistics. https://www.bls.gov/oes/.

[32] Reeves A., Loopstra R., Tarasuk V. (2021). Wage-Setting Policies, Employment, and Food Insecurity: A Multilevel Analysis of 492 078 People in 139 Countries. Am. J. Public Health.

[33] Linnan L., Arandia G., Bateman L., Vaughn A., Smith N., Ward D. (2017). The Health and Working Conditions of Women Employed in Child Care. Int. J. Environ. Res. Public Health.

[34] Kanervisto M., Vasankari T., Laitinen T., Heliövaara M., Jousilahti P., Saarelainen S. (2011). Low Socioeconomic Status Is Associated with Chronic Obstructive Airway Diseases. Respir. Med..

[35] Tovar A., Vaughn A.E., Grummon A., Burney R., Erinosho T., Østbye T., Ward D.S. (2017). Family Child Care Home Providers as Role Models for Children: Cause for Concern?. Prev. Med. Rep..

[36] Loh I.H., Oddo V.M., Otten J. (2020). Food Insecurity Is Associated with Depression among a Vulnerable Workforce: Early Care and Education Workers. Int. J. Environ. Res. Public Health.

[37] Swindle T.M., Ward W.L., Bokony P., Whiteside-Mansell L. (2018). A Cross-Sectional Study of Early Childhood Educators’ Childhood and Current Food Insecurity and Dietary Intake. J. Hunger Environ. Nutr..

[38] Ali U., Herbst C.M., Makridis C.A. (2021). The Impact of COVID-19 on the U.S. Child Care Market: Evidence from Stay-at-Home Orders. Econ. Educ. Rev..

[39] Sonnier-Netto L., Cope H., Falgoust T., Oakey-Frost R., Lewis R. (2020). The Ongoing Impacts of COVID-19 on Louisiana Child Care Providers: Statewide Survey Results from April 13–April 20, 2020.

[40] Jacobs K., Henchy G. (2020). Child and Adult Care Food Program Participation and Reimbursement during the COVID-19 Pandemic: Analysis of Preliminary USDA Data.

[41] He Y. (2023). Impact of Coronavirus Disease 2019 on Food Security in Early Childhood. Curr. Opin. Pediatr..

[42] Sethi S., Johnson-Staub C., Robbins K.G. (2020). An Anti-Racist Approach to Supporting Child Care Through COVID-19 and Beyond.

[43] Austin L.J.E., Edwards B., Chávez R., Whitebook M. (2019). Racial Wage Gaps in Early Education Employment.

[44] Kalinowski J., Wurtz H., Baird M., Willen S.S. (2022). Shouldering the Load yet Again: Black Women’s Experiences of Stress during COVID-19. SSM—Ment. Health.

[45] Rogers T.N., Rogers C.R., VanSant-Webb E., Gu L.Y., Yan B., Qeadan F. (2020). Racial Disparities in COVID-19 Mortality Among Essential Workers in the United States. World Med. Health Policy.

[46] Heflin C.M., Siefert K., Williams D.R. (2005). Food Insufficiency and Women’s Mental Health: Findings from a 3-Year Panel of Welfare Recipients. Soc. Sci. Med..

[47] Heflin C.M., Ziliak J.P. (2008). Food Insufficiency, Food Stamp Participation, and Mental Health*. Soc. Sci. Q..

[48] Herman D.R., Westfall M., Bashir M., Afulani P. (2024). Food Insecurity and Mental Distress Among WIC-Eligible Women in the United States: A Cross-Sectional Study. J. Acad. Nutr. Diet..

[49] Leung C.W., Epel E.S., Willett W.C., Rimm E.B., Laraia B.A. (2015). Household Food Insecurity Is Positively Associated with Depression among Low-Income Supplemental Nutrition Assistance Program Participants and Income-Eligible Nonparticipants. J. Nutr..

[50] US Department of Agriculture, Economic Research Service Food Security in the United States: Measurement. https://www.ers.usda.gov/topics/food-nutrition-assistance/food-security-in-the-u-s/measurement/.

[51] Skouteris H., Green R., Chung A., Bergmeier H., Amir L.H., Baidwan S.K., Chater A.M., Chamberlain C., Emond R., Gibbons K. (2022). Nurturing Children’s Development through Healthy Eating and Active Living: Time for Policies to Support Effective Interventions in the Context of Responsive Emotional Support and Early Learning. Health Soc. Care Community.

[52] Food and Nutrition Service, United States Department of Agriculture Child and Adult Care Food Program. https://www.fns.usda.gov/cacfp.

[53] Dev D.A., Hillburn C., Luxa J., Bauer K.W., Lessard L., Cotwright C., Tovar A. (2023). Illuminating Child and Adult Care Food Program Partnerships That Improved Food Access and Waiver Utilization for Feeding Young Children in Early Care and Education Programs During COVID-19: A Qualitative Study. J. Acad. Nutr. Diet..

[54] Lee D.L., Homel Vitale E., Marshall S.K.-D., Hecht C., Beck L.T., Ritchie L.D. (2022). Child and Adult Care Food Program Participation Benefits, Barriers and Facilitators for Independent Child Care Centers in California. Nutrients.

[55] Makelarski J.A., Abramsohn E., Benjamin J.H., Du S., Lindau S.T. (2017). Diagnostic Accuracy of Two Food Insecurity Screeners Recommended for Use in Health Care Settings. Am. J. Public Health.

[56] Hutton N.S., McLeod G., Allen T.R., Davis C., Garnand A., Richter H., Chavan P.P., Hoglund L., Comess J., Herman M. (2022). Participatory Mapping to Address Neighborhood Level Data Deficiencies for Food Security Assessment in Southeastern Virginia, USA. Int. J. Health Geogr..

[57] National Academies of Sciences, Engineering, and Medicine Mitigating the Effects of Pandemic on Early Child Care and Education Providers—New Consultation. https://www.nationalacademies.org/news/2022/01/mitigating-the-effects-of-pandemic-on-early-child-care-and-education-providers-new-consultation.

